# Associations between Common Variants in Iron-Related Genes with Haematological Traits in Populations of African Ancestry

**DOI:** 10.1371/journal.pone.0157996

**Published:** 2016-06-22

**Authors:** Wanjiku N. Gichohi-Wainaina, Toshiko Tanaka, G. Wayne Towers, Hans Verhoef, Jacobien Veenemans, Elise F. Talsma, Jan Harryvan, Mark V. Boekschoten, Edith J. Feskens, Alida Melse-Boonstra

**Affiliations:** 1 Division of Human Nutrition, Wageningen University, Wageningen, the Netherlands; 2 International institute of Tropical Agriculture (IITA), Dar es Salaam, Tanzania; 3 Translational Gerontology Branch, National Institute on Aging, Baltimore, MD, United States of America; 4 Centre of Excellence for Nutrition, North-West University (Potchefstroom campus), Potchefstroom, South Africa; 5 Cell Biology and Immunology Group, Wageningen University, Wageningen, The Netherlands; 6 Medical Research Council (MRC) International Nutrition Group, London School of Hygiene and Tropical Medicine, London, United Kingdom; 7 Medical Research Council Banjul, The Gambia; 8 Laboratory for Microbiology and Infection Control, Amphia Hospital, Breda, The Netherlands; 9 Department of Medical Microbiology and Immunology, Admiraal De Ruyter Hospital, Goes The Netherlands; 10 HarvestPlus, International Center for Tropical Agriculture (CIAT), Cali, Colombia; 11 Nutrition, Metabolism and Genomics group, Division of Human Nutrition, Wageningen University, Wageningen, The Netherlands; Vanderbilt University Medical Center, UNITED STATES

## Abstract

**Background:**

Large genome-wide association (GWA) studies of European ancestry individuals have identified multiple genetic variants influencing iron status. Studies on the generalizability of these associations to African ancestry populations have been limited. These studies are important given interethnic differences in iron status and the disproportionate burden of iron deficiency among African ancestry populations.

**Methods:**

We tested the associations of 20 previously identified iron status-associated single nucleotide polymorphisms (SNPs) in 628 Kenyans, 609 Tanzanians, 608 South Africans and 228 African Americans. In each study, we examined the associations present between 20 SNPs with ferritin and haemoglobin, adjusting for age, sex and CRP levels.

**Results:**

In the meta analysis including all 4 African ancestry cohorts, we replicated previously reported associations with lowered haemoglobin concentrations for rs2413450 (β = -0.19, P = 0.02) and rs4820268 (β = -0.16, P = 0.04) in *TMPRSS6*. An association with increased ferritin concentrations was also confirmed for rs1867504 in *TF* (β = 1.04, P = <0.0001) in the meta analysis including the African cohorts only.

**Conclusions:**

In all meta analyses, we only replicated 4 of the 20 single nucleotide polymorphisms reported to be associated with iron status in large GWA studies of European ancestry individuals. While there is now evidence for the associations of a number of genetic variants with iron status in both European and African ancestry populations, the considerable lack of concordance highlights the importance of continued ancestry-specific studies to elucidate the genetic underpinnings of iron status in ethnically diverse populations.

## Introduction

Body iron status is influenced by a combination of genes as well as environmental factors such as diet, blood loss, pregnancy, alcohol intake and infections [1, 2]. In order to maintain iron levels within normal ranges and thus prevent iron excess or iron deficiency, iron homeostasis has evolved as a complex trait and a tightly coordinated process of interaction between genes and environmental factors. Whether genetic influences on iron metabolism may influence iron status at the public health level, is a subject of recent interest. This is of interest since segments of populations that are genetically prone to iron deficiency can be specifically targeted with interventions.

Understanding the genetic underpinnings of low iron status is particularly relevant for African ancestry populations, who experience a disproportionately high burden of low iron status. In particular, it has been observed that in the US, African-Americans have lower haemoglobin concentrations and serum transferrin saturation (TS), and higher serum ferritin (SF) concentrations compared to individuals of European Ancestry [3]. Furthermore, according to WHO regional prevalence estimates, Africa has the highest proportion of individuals affected by anaemia worldwide [4].

Previous studies on iron status report heritability estimates ranging from of 20% to 30%, supporting a genetic component to iron status [5, 6]. Genome-wide association (GWA) studies have identified common variants that are associated with iron status in largely European ancestry populations [7–10]. It is questionable however whether these findings can be generalized to African ancestry populations. Given the greater levels of linkage disequilibrium among European-ancestry individuals [11], it is expected that index single nucleotide polymorphisms (SNPs) tag larger regions of the genome than they would among African ancestry individuals. Therefore index SNPs in European populations may not necessarily tag causal variants within the African population. Moreover, African populations are exposed to very different environmental factors that further influence iron status. Recently, genetic studies on iron status have been conducted in the African American population [12–16]. This population has varying degrees of admixture that may complicate transferability of these results to African populations [17]. Conclusive inferences about the potential generalization of previous findings to African ancestry populations have been precluded by the lack of data from African cohorts. In part this has been due to the fact that it is challenging to find African cohorts which can be utilized in genetic epidemiologic studies. In the present study, we utilized data from four African ancestry cohorts that were initiated with different primary hypotheses but had collected the necessary phenotypic and genetic data to address the question regarding the genetic architecture of iron metabolism. We investigated 20 SNPs previously reported to be associated with iron status in GWA studies [7, 8, 10, 14, 18] with haematological indicators in several African ancestry populations.

## Methods

### Study area and population

Four cohorts of African ancestry were selected; from Kenya (n = 628), Tanzania (n = 609); South Africa (n = 686) and the United States of America (n = 228). The four cohorts differed largely in age, gender and burden of inflammation as described in more detail below.

#### Kenyan cohort

Samples were obtained from both a cross sectional study and from the baseline data of a randomized trial *(Cassavita study)* designed to assess the efficacy of bio-fortified cassava to improve vitamin A deficiency [19, 20]. The cross sectional study was conducted in June 2010. The randomized trial (*Cassavita study*) was conducted from May until November 2012 in 3 primary schools in Kibwezi District, Kenya. Haematological markers were measured prior to the start of the trial. As determined by self-report in both studies children were of Akamba (Bantu) ancestry. Blood samples for the cross-sectional study were collected from children aged 6–12 years (n = 375) who were located in the Kibwezi and Makindu Districts in Eastern Kenya, which is a low malarial transmission area [21]. Blood samples for the randomized trial were collected from children aged 5–13 years (n = 342) in three primary schools located in the Kibwezi District. Children with lowest retinol-binding protein concentrations were included in the trial to select for those with the lowest vitamin A status. All subjects (for both the trial and cross sectional study) were apparently healthy and without fever (ear drum temperature < 37.5°C) upon examination by the research physician and children with inflammation (C-reactive protein concentration > 8mg/L), low haemoglobin concentrations (<70 g/L), or *Plasmodium* infection were excluded.

The cross sectional study as well as the trials were approved by ethical committees in Kenya and the Netherlands (ClinicalTrials.gov:NCT01614483). Written informed consent was obtained from parents or guardians, and assent from children.

#### Tanzanian cohort

Children in this cohort were part of a randomized placebo controlled trial *(MACH study)* [22]. Data were collected between February 2008 and March 2009. Haematological markers as were measured in samples collected prior to the start of the trial and ancestry was self-reported. People in this area originally belonged to the Wazigua and Wabondei Bantu tribes, but settlement of migrant plantation workers in the past has resulted in the admixture of many tribes with different origins. Samples were obtained from children aged 6–60 months (n = 612) with height-for-age z-score ≤ –1.5 SD in a rural area of Handeni District in North-Eastern Tanzania, which has intense year-round *P*. *falciparum* transmission. Children with severe wasting (weight-for-age z-score < –3 SD), haemoglobin concentration < 70 g/L, *Plasmodium* infection or signs of chronic illness were excluded.

The study was approved by the Ethical Review Committee of Wageningen University, The Netherlands (ClinicalTrials.gov: NCT00623857) and the National Health Research Ethics Review sub-Committee, Dar es Salaam, Tanzania. Written individual informed consent was obtained from parents or primary caretakers.

#### South African cohort

This study population was a subset of Tswana speaking pre- and post-menopausal women (n = 686) enrolled as part of the South African arm of the Prospective Urban and Rural Epidemiological (PURE) study. The PURE study began in 2005. Haematological measures utilized in this study were collected in a follow up study conducted in 2010. The participants were from the North-West Province in South Africa, aged 35 years or older and were mainly of Tswana (Bantu) ancestry as determined by self-report. Women were only eligible for this sub-study if they were apparently healthy, not pregnant, non-lactating and were at the time of blood collection not diagnosed with any chronic disease or taking drugs for any chronic disease. Additionally, we included only participants with all outcome measures determined and who were HIV negative. HIV testing was undertaken with the appropriate pre- and post-test counselling as required by South African law. This study was approved by the Ethics Committee of the North-West University and signed informed consent forms were obtained from all participants.

#### African American cohort

This cohort consisted of participants from the Baltimore Longitudinal Study on Aging (BLSA). The BLSA is a population-based study aimed at evaluating contributors of healthy aging in the older population residing predominantly in the Baltimore-Washington DC area [23]. Started in 1958, participants aged 17–94 years were examined every one to four years depending on their age. Currently, there are approximately 1,100 active participants enrolled in the study. For the present study, 228 subjects of African ancestry with complete data on both genetic and haematological traits were available and were used for the analysis. The BLSA has continuing approval from the Institutional Review Board (IRB) of Medstar Research Institute, Johns Hopkins, Bayview Medical Centre, and the University of Maryland. Additionally, BLSA study participants provide written informed consent to participate in both direct and ancillary studies related to their collected data.

### Iron related measurements

#### Kenyan and Tanzanian cohorts

For the cross sectional study (Kenyan cohort), serum ferritin concentrations were determined by enzymatic immunoassay (Ramco Laboratories, Stafford Texas) and haemoglobin concentration (Hb) was assessed using a hematology analyzer (Celltac-α, MEK-6410K, Nihon Kohden Corporation, Tokyo, Japan). Serum concentrations of CRP were determined by immunoturbidometric assay on a Cobas Integra 800 system (Roche Diagnostics, Mannheim, Germany). For the Kenyan randomized trial as well as the Tanzanian cohort, concentrations of ferritin and concentrations of CRP were measured on a Beckman Coulter UniCel DxC 880i analyzer as per manufacturer’s instructions.

#### South African cohort

Serum ferritin was determined using an enzyme immunoassay procedure (Ramco Laboratories, Inc, Stafford Texas). Hb concentrations were determined spectrophotometrically from whole blood using the Hemocue^®^ HB201 haemoglobin meter (Hemocue AB, Angelholm Sweden). Serum concentrations of CRP were measured from serum via a particle-enhanced immunoturbidometric assay using Sequential Multiple Analyser Computer (SMAC) using the Konelab20iTM auto analyser (Thermo Fisher Scientific Oy, Vantaa, Finland). Whole blood was used for the determination of HIV status making use of the First Response (PMC Medical, India) rapid HIV Card Test. If tested positive, the test was repeated with the Pareeshak (BHAT Bio-tech India) card test.

#### African American cohort

Serum ferritin was measured using an immunoassay type two-stage sandwich method using two antiferritin antibodies (Advia Centaur, Bayer). Haemoglobin was assessed using SYSMEX XE-series (Sysmex Corporation). Serum concentrations of CRP were measured using the BNII nephelometer from Dade Behring utilizing a particle enhanced immunonepholometric assay.

### SNP selection, genotyping and quality control procedures

#### SNP selection

To set up the assay for genotyping it was required to develop a list of reference SNPs (rs) numbers from Genbank to be included in the analysis. This list was set up by performing a literature search of Pubmed, Sciencedirect, Ebscohost, HUGE navigator and Google Scholar using the following keywords:

1. Iron, ferritin, transferrin
2. Anaemia
3. Haemochromatosis
4. Human
5. SNP, Polymorphism, Mutation
6. Genetic

All abstracts were scanned for appropriateness of use, that is, association or linkage of a SNP to the haematological parameters or key biomarkers of iron status (Hb, SF, serum transferrin receptor (sTfR) and body iron (BI)). If appropriate, the reference SNP (rs) number of the polymorphism was recorded and included in the list to be sent to Illumina for verification of use in the array. Based on this, we selected 32 SNPs for genotyping. Once SNPs were selected, genotyping was performed for the Kenyan, Tanzanian and South African cohorts. These data were complemented with the data we obtained from the African American cohort in the BLSA.

#### Genotyping and quality control procedures

For the Kenyan, Tanzanian and South African cohorts, all 32 rs numbers were submitted to Illumina Technical Support for evaluation using the Assay Design Tool. Each SNP was scored (varying from 0 to 1) by the same, based on its compatibility for use in a GoldenGate genotyping assay. SNPs with a score above 0.4 were chosen for genotyping. No SNP fell out based on these criteria. Genotyping for Kenyan (efficacy trial only), South African and Tanzanian cohorts was conducted using the BeadXpress^™^ platform. Once genotyping was performed, several quality control procedures were applied. Lack of a detectable signal after analysis was designated as a failed SNP. SNP clustering was also assessed visually to determine success of genotyping. SNPs with a GenCall Score of >0.5 and samples with a call rate of ≥0.95 were included in the final analysis. Genotyping was performed for only three SNPs in *TMPRSS6* (rs2413450, rs4820268, rs228918), for the Kenyan pilot study samples using Applied Biosystems TaqMan Assays. Data obtained with this method were merged with the data from the Kenyan efficacy trial. Eventually, we considered only 20 SNPs that had passed all quality control procedures in all four cohorts for association analyses (S1 Table).

For the African American cohort, genome-wide genotyping was conducted using the Illumina Infinium HumanHap 550K. Genotyping was completed for 284 subjects, and imputation of ~2.5 million HapMap SNPs was conducted using 501,764 SNPs that passed quality control (minor allele frequency > 1%, genotyping completeness > 99%, and Hardy Weinberg-equilibrium > 0.0001). Imputation was conducted with MACH using the HapMap YRI sample (HapMap data release rel#23a NCBI B36) as a reference. The candidate iron-homeostasis SNPs that were either directly genotyped or imputed (with an imputation quality of greater than r^2^>0.3) were used for the present analysis.

### Statistical analyses

Outcome variables (haemoglobin and serum ferritin concentrations) were determined to be continuous quantitative traits. Anaemia was defined using different cut offs for each cohort as cut offs are age dependent [24]. Cut offs were as follows: Hb concentration <11.5 g/dL (Kenyan cohort), <11 g/dL (Tanzanian cohort), <12 g/dL for the South African and African American cohorts, while iron deficiency was defined as serum ferritin concentration <15μg/L for Kenyan, South African and African American samples, and plasma ferritin concentration <12μg/L for Tanzanian samples. Log_10_ transformations were applied to the ferritin outcomes to improve their fit to the normal distribution. Results from associations involving ferritin were then presented as back-transformed data. Potential disturbances in the distributions of the genotypes were tested for deviation from the assumptions of Hardy-Weinberg equilibrium (HWE) by using the Chi-square test. To compare minor allele frequencies (MAFs) across cohorts, a spider web graph was generated using Microsoft Excel (Version 2010), including European ancestry populations from the 1000 Genomes Project [20]. Linear regression was used to assess associations between SNPs and outcome measures. For association testing, genotypes were coded as 0, 1, and 2 reflecting the number of copies of the minor allele (additive genetic model), while imputed genotypes were coded using fractional counts between 0 and 2 according to the estimated number of copies of each minor allele. All associations were adjusted for age and gender. Additionally, associations involving ferritin concentrations were adjusted for inflammation by including CRP concentrations as a covariate in the model. Statistical analyses were performed using version 20 of the Statistical Package for Social Sciences (SPSS Inc Chicago III) or SAS version 9.2 (Cary, NC). All reported P-values were two-tailed, and adjustment for multiple testing was performed using the false discovery rate (FDR) method. The R program for statistical computing version 2.15.2 [25] was used to perform all meta-analyses and chi square tests conducted to test for heterogeneity between studies. We performed the meta analyses in order to increase the sample size and obtain a summary statistic. Comparisons of effect sizes and directions were made within and across populations, and meta-analyses were performed to generate summary effect estimates with and without including the African American cohort. Statistical significance for all analyses was defined as α < 0.05, while the q values cut-off was set at 0.10.

## Results

There were large differences in age between the cohorts, as the studies from Kenya and Tanzania were pre- and school going children, while the studies from South Africa and the United States were of adults (Table 1). With the exception of the South African cohort which was all women, the sex distribution was approximately equal. Prevalence of inflammation was below 5% in the Kenyan and African American cohort and 33% and 43% in the Tanzanian and South African cohort, respectively. Iron deficiency was highest in the Kenyan cohort (37%) and lowest in the African American cohort (3%). Anaemia was highest in the Tanzanian cohort (32%) and lowest in the Kenyan cohort at (7%).

**Table 1:** General characteristics of the study participants in the four cohorts.

	*n*	Kenya	*n*	Tanzania	*N*	South Africa	*N*	USA (African American)
Age, y	628	9.0 (2.1)	609	2.7 (1.3)	686	50.0 (10.0)	228	67.4 (11.0)
Gender (% female)	628	52	609	51	686	100	228	59
Haemoglobin (g/dL)	626	13.9 (1.6)	607	10.3 (12.8)	481	13.8 (1.6)	228	13.0 (1.4)
Ferritin (μg/L)^$^	623	17.8 (17.0,18.6)	603	31.6 (31.6, 39.8)	686	85.1 (77.6, 93.3)	221	103.4 (96.6)
Inflammation (%)	620	3	609	33	290	43	228	5
Anaemia (%)	626	7	609	32	481	8	227	19
Iron deficiency (%)	623	37	606	18	686	11	227	3

^$^ Values are geometric means (confidence intervals) or otherwise means (SD); Cut-offs for inflammation: C-reactive protein>5mg/L; Iron deficiency: Serum ferritin <15μg/l (Kenyan, South African and African American cohorts), and Plasma ferritin <12μg/L (Tanzanian cohort); Anaemia: Haemoglobin <11.5 g/dL (Kenyan cohort), <11 g/dL (Tanzanian cohort), or <12 g/dL (South African and African American cohorts).

**Fig 1:**
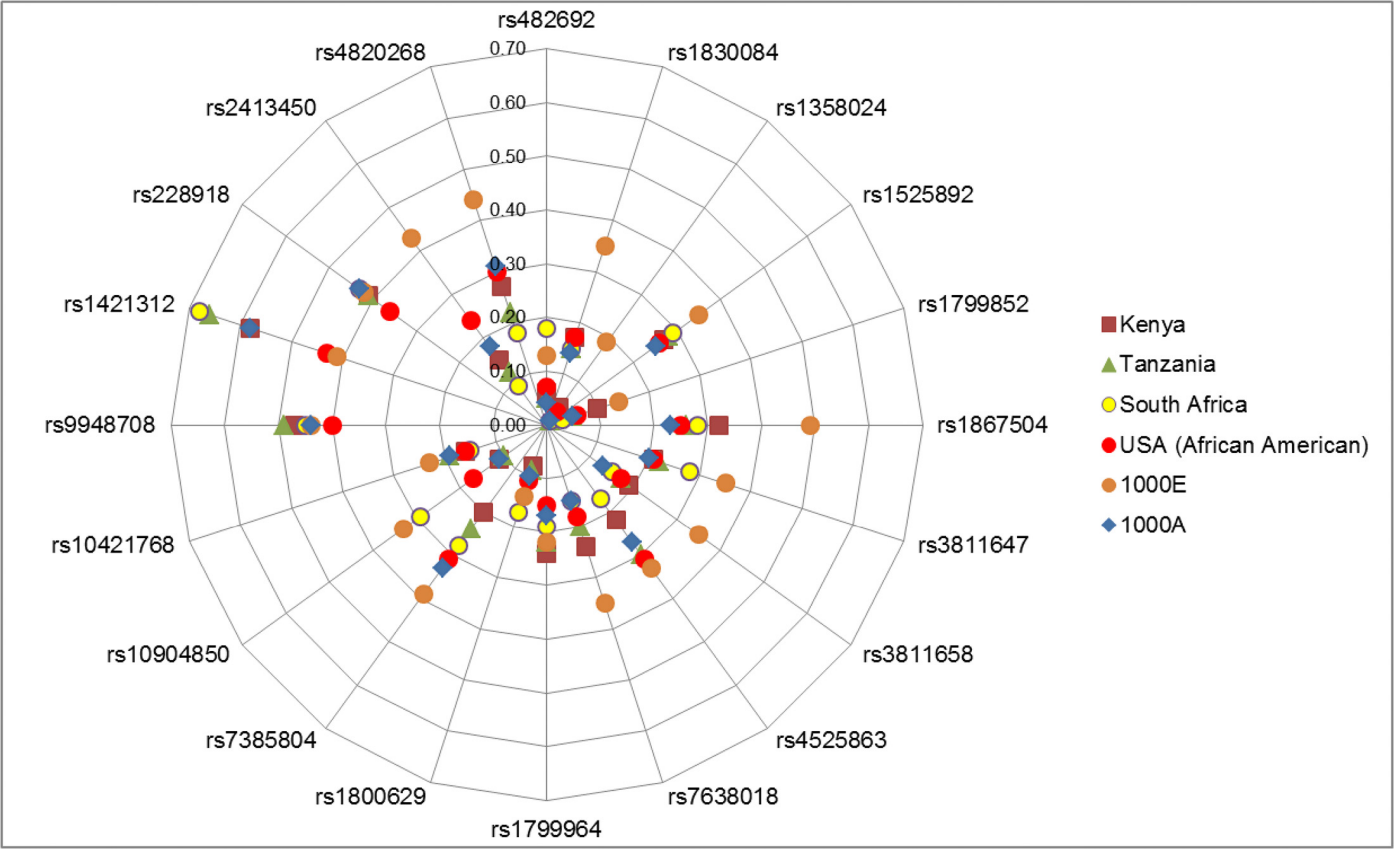
Minor allele frequency of common genetic variants related to iron metabolism in four African ancestry populations in comparison to the African and European population in the 1000 Genomes Project database. MAF 1000 A, Minor allele frequency for Africans-1000 Genomes Project; MAF 1000 E, Minor allele frequency for Europeans- 1000 Genomes Project.

In comparing minor allele frequencies across cohorts, the SNPs genotyped for the present study were generally observed to be more common in European ancestry cohorts as reported in the 1000 Genomes Project database (Fig 1). For example, for the commonly investigated rs4820268 SNP in the *TMPRSS6* gene, the frequency of the allele associated with lower iron status was 27%, 22%, 18%, and 30% in the Kenyan, Tanzanian, South African and African American cohorts, respectively. According to the 1000 Genomes Project database, this is comparable to the 31% reported for Africans, but substantially lower than the 41% reported for Europeans. Similarly, for rs2413450 (*TMPRSS6*), MAFs were ~12% in the Kenyan, Tanzanian and South African cohorts and 24% in the African American cohort, which is comparable to the MAF for Africans (17%), but again lower than that for Europeans (42%) reported in the 1000 Genomes Project database. On the other hand, rs1421312 (*TMPRSS6*) was more common in the African cohorts (Kenya, 58%; Tanzania, 66%; South Africa, 68%; 1000 Genomes, 58%) compared to the Europeans (41%). For the same SNP (rs1421312), the African American cohort had a MAF of 43%.

In linear regression analyses with haemoglobin concentrations as the dependent outcome the minor allele (A) of rs10904850 in *CUBN* was associated with lower Hb concentrations of 0.38 g/dL (P = 0.02). Lower Hb concentrations were also observed for the minor allele (G) of rs4820268 in *TMPRSS6* (0.28 g/dL; P = 0.01) in the Kenyan children (Table 2). Conversely, the minor allele (A) of rs10421768 in *HAMP* was positively associated with increased Hb concentrations of 0.37 g/dL (P *=* 0.01) in the Kenyan population.

**Table 2:** Associations of single nucleotide polymorphism with haemoglobin in four cohorts of African ancestry with meta-analyses.

		Ke		Tz		SA^†^		Met_African			AA		Met_All		
SNP	Minor Allele	β (se)	P	β (se)	P	β (se)	P	β (se)	P	P (Het)	β (se)	P	β (se)	P	P (Het)
***TF***															
rs3811658	T	0.01(0.13)	0.95	1.57(0.99)	0.11	0.10(0.04)	0.49	**0.36(0.04)**	**0.02**	0.26	0.08(0.16)	0.61	**0.09(0.03)**	**0.01**	0.45
***CUBN***															
rs10904850	A	**-0.38(0.16)**	**0.02**	0.91(1.19)	0.44	0.07(0.11)	0.56	-0.11(0.10)	0.14	0.05	0.03(0.16)	0.86	-0.07(0.14)	0.63	0.10
***HAMP***															
rs10421768	A	**0.37(0.19)**	**0.01**	0.46(0.94)	0.62	-0.01(0.15)	0.93	0.19(0.18)	0.29	0.17	0.14(0.12)	0.12	0.17(0.10)	0.09	0.30
***TMPRSS6***															
rs2413450	A	-0.20(0.12)	0.10	-0.65(1.13)	0.57	-0.23(0.18)	0.20	**-0.23(0.10)**	**0.02**	0.93	-0.09(0.14)	0.55	**-0.19(0.08)**	**0.02**	0.84
rs4820268	G	**-0.28(0.11)**	**0.01**	-0.26(0.87)	0.76	-0.05(0.14)	0.74	**-0.18(0.07)**	**0.01**	0.43	-0.09(0.13)	0.48	**-0.16(0.07)**	**0.04**	0.55

Differences in haemoglobin concentrations are in g/dL. SNP = Single nucleotide polymorphism; Ke = Kenyan cohort; Tz = Tanzanian cohort; SA = South African cohort; AA = African American cohort; Met-African = meta analyses of beta and SE values from African cohorts; Met_All = meta analyses of beta and SE values from all cohorts. Results are presented as mean (SE) change per copy of the minor allele from regression analysis using an additive genetic model and with adjustment for age and sex.

^†^ Values not adjusted for gender as all participants were women. P (Het) = Cochran’s Q to measure heterogeneity between cohorts with a value of 0.10 as a cut-off for significance. Values in bold are significant before FDR correction for multiple testing. Explained variance ranged from <1% to 3%, with the largest explained variance being 3% for rs10421768 in the Kenyan population.

After meta-analyses of data from all three African cohorts, the minor allele (T) of rs3811658 in *TF* was associated with a higher Hb concentration of 0.36 g/dL (P = 0.02) while minor alleles of both rs2413450 (A) and rs4820268 (G) in *TMPRSS6* were associated with lower Hb concentrations of -0.23 g/dL (P = 0.02) and -0.18 g/dL (P = 0.01) respectively. The associations of rs3811658, rs2413450 and rs4820268 with Hb remained significant and in the same direction after including the African American cohort in the meta-analysis. There was no heterogeneity between studies for these SNPs.

The minor allele (A) of rs1799852 in *TF* was associated with lower SF concentrations in the South African cohort (-0.62 μg/L, P = 0.01). On the other hand the minor allele (A) of rs1867504 also in *TF* was associated with increased serum ferritin concentrations (1.04 μg/L, P = 0.06), in the meta analyses of data from the three African populations.

The minor alleles (C) of rs7385804 in *TFR2* was associated with decreased serum ferritin concentrations in the Kenyan population of -0.86 μg/L (P = 0.02). Lowered serum ferritin concentrations were also observed for rs1800629 (A) in *TNF* (-0.76 μg/L, P = 0.03, respectively) within the African American cohort (Table 3). No significant associations were observed in the meta analyses including the African American cohort though associations of the minor alleles of rs1525892 (P = 0.05), rs1867504 (P = 0.06) and rs 3811647 (P = 0.05) in *TF* and rs10421768 in *HAMP* (P = 0.06) with ferritin concentrations were nearly significant (S1 Table). Complete information on associations of all 20 SNPs with concentrations of haemoglobin and ferritin can be found in the supplementary table (S1 Table).

**Table 3:** Associations of single nucleotide polymorphism with ferritin in four cohorts of African ancestry with meta-analyses.

		Ke		Tz		SA^†^		Met_African			AA		Met_All		
SNP	Minor Allele	β (se)	P	β (se)	P	β (se)	P	β (se)	P	P (Het)	β (se)	P	β (se)	P	P (Het)
***TF***															
rs1799852	A	-0.98(1.09)	0.80	1.20(1.15)	0.18	**-0.62(1.2)**	**0.01**	-0.15(0.68)	0.41	0.34	1.23(1.17)	0.21	0.18(0.60)	0.76	0.37
rs1867504	A	1.04(1.06)	0.62	1.05(1.07)	0.52	1.03(1.08)	0.69	**1.04(0.06)**	0.00*	1.00	1.05(1.10)	0.62	1.04(0.54)	0.06	1.00
***TFR2***															
rs7385804	C	**0.86(1.07)**	**0.02**	0.95(1.08)	0.48	1.15(1.07)	0.06	0.99(0.62)	0.11	0.98	1.07(1.10)	0.52	1.01(0.54)	0.06	1.00
***TNF***															
rs1800629	A	1.02(1.10)	0.85	1.05(1.12)	0.62	1.03(1.09)	0.74	1.03(0.64)	0.10	1.00	**-0.76(1.15)**	**0.03**	0.61(0.56)	0.27	0.60

Ferritin concentrations are in μg/L. Ferritin values are Log_10_ back transformed data. SNP = Single nucleotide polymorphism; Ke = Kenyan cohort; Tz = Tanzanian cohort; SA = South African cohort; AA = African American cohort; Met-African = meta analyses of beta and SE values from African cohorts; Met_All = meta analyses of beta and SE values from all cohorts. Results are presented as mean (SE) change per copy of the minor allele from regression analysis using an additive genetic model and with adjustment for age and sex.

^†^ Values not adjusted for gender as all participants were women.

* = P <0.0001; P (Het) = Cochran’s Q to measure heterogeneity between cohorts with a value of 0.10 as a cut-off for significance. Values in bold are significant before FDR correction for multiple testing. Explained variance ranged from < 1% to 3%, with the largest explained variance being 3% for rs10421768 in the Kenyan population.

None of the associations for either Hb or SF as outcome measures were significant after FDR correction for multiple testing.

## Discussion

We report here one of the few candidate gene association studies focussing on the genetics of iron status in various African ancestry populations. In the meta analyses involving all four cohorts, we have observed small differences in Hb concentrations between genotype groups: slightly lower Hb concentrations were associated with the minor alleles A of rs2413450 and G of rs4820268, both in the *TMPRSS6 gene* and higher Hb concentrations involving the minor allele T in rs3811658 in the *TF* gene. In similar investigations involving ferritin concentrations, the minor allele A of rs1867504 in *TF* was associated with higher ferritin concentrations. Additionally, there were marginally significant associations with higher ferritin concentrations for the minor alleles of rs1525892, and rs3811647 in *TF*, rs7385804 in *TFR2* and rs10421768 in *HAMP*. We further observed that many of the candidate SNPs genotyped in our cohorts, were more common in the European ancestry cohort in the 1000 Genomes Project as compared to our African ancestry cohorts.

The most consistent association in terms of direction as well as statistical significance was observed for rs2413450 and rs4820268 in the *TMPRSS6* gene, both associated with lower Hb concentration. Similar observations have previously been made within populations of European and Asian ancestry [7, 10, 26, 27]. The exact mechanism through which *TMPRSS6* action is exerted is still under investigation. It has generally been hypothesised that *TMPRSS6* polymorphisms affect hepcidin antimicrobial peptide (HAMP) transcription, thereby influencing hepcidin concentrations in response to systemic iron concentrations [28–30]. However, two recent studies [31, 32] did not confirm an intermediate role for hepcidin in the SNP-iron status parameter associations. Currently, it is proposed that matriptase-2 could regulate hepcidin expression by cleaving HJV to decrease BMP-SMAD signalling [33].

Several *TF* variants (rs3811658, rs1867504 and rs1799852) were also significantly associated with various iron status measures. In contrast to what is known from other studies, we observed associations between rs3811658 in the *TF* gene with higher Hb concentrations. This SNP is in linkage disequilibrium with rs3811647 that has previously been negatively associated with serum iron, body iron, transferrin saturation and serum ferritin concentrations and positively associated with serum transferrin concentrations [9, 34]. Also, the SNP has been associated with increased risk of iron deficiency in a population of men and women of European descent [35]. As we would have expected to see negative associations between rs3811658 with haemoglobin concentrations, our finding requires further investigation.

The variant rs1867504 in *TF* has previously been associated with increased serum transferrin concentrations in a cohort of European ancestry [9] but not with serum ferritin as seen in our study. It is possible that this variant has pleiotropic effects on ferritin as has previously been observed for other *TF* SNPs [9].

Previously, three variants in *TF* (rs3811647, rs1799852 and rs2280673) plus the HFE C282Y mutation have been observed to explain approximately 40% of genetic variation in serum transferrin [9]. We only observed a significant association of rs1799852 with serum ferritin in the South African cohort. Furthermore, we did not have information on rs2280673 and HFE C282Y. We therefore could not replicate this previously observed association.

*TF* is a crucial biological carrier of iron in blood plasma[36]. Severe mutations in the transferrin gene lead to atransferrinaemia [37] and have been determined to cause microcytic anaemia accompanied by hepatic accumulation of iron in humans [38] and mice [39]. Further investigations into the influence of various *TF* variants on haematological traits are therefore required.

A large portion of systemic iron deficiency undoubtedly results from non-genetic factors. Environmental factors such as diets low in bioavailable iron [40] as well as a high inflammation burden [41], are probably the foremost factors predisposing to iron deficiency. High inflammation burden causes iron to be retained in the reticuloendothelial system, resulting in iron being unavailable for erythropoiesis [41]. Diets in African populations are typically low in bioavailable iron [42]. We corrected for dietary iron intake in the African American as well as South African cohorts (not presented), but the difference between the crude and adjusted regression models was negligible. To account for other environmental and individual influences on iron status, we adjusted our regression models for major biological confounders that are known to affect Hb and ferritin concentrations including age, gender [43] and the inflammatory acute phase protein CRP [41] to increase precision of our estimates. Age and gender are known to influence iron status [44, 45]. Although we corrected the associations with serum ferritin as the outcome for CRP, we may not have captured the inflammation at later convalescence.

It is noteworthy that we did observe population specific significant associations that were not replicated in all cohorts. This was particularly observed in the Kenyan cohort that consisted of school going children (mean age 9.0 years). In this cohort we specifically highlight the association of the A allele of rs10421768 in *HAMP* with Hb and the C allele of rs7385804 in *TFR2*.

The HAMP gene encodes hepcidin, which is a 25-amino acid peptide produced and secreted mainly by hepatocytes and is a major regulator of systemic iron homeostasis [33]. As far as we know, no functional studies have investigated the effect of rs10421768 on dysregulation of hepcidin expression in humans. Based on our data, we speculate that this SNP down regulates *HAMP*, thereby increasing the amount of iron in circulation available for erythropoiesis. Indeed, we observed rs10421768 in *HAMP* to be consistently associated with higher serum ferritin concentrations across all study populations (S1 Table).

The SNP rs7385804 has previously been associated with reduced serum iron, serum transferrin and transferrin saturation concentrations in a cohort of elderly Chinese women [26]. *TFR2* polymorphisms have also been reported to be associated with red blood cell count and mean cell volume [46–48]. In terms of function, *TFR2* is believed to be involved in the hepcidin regulation pathway [49]. The fact that SNPs directly linked to hepcidin were observed to be associated with various iron status measures underscores the key role that hepcidin could play in determining iron status in the Kenyan cohort. The children in this cohort did not reside in a malaria endemic area and based on CRP concentrations did not have a high prevalence of inflammation, both of which are likely to impact significantly on hepcidin concentrations. Hepcidin however responds to signals from diverse physiological processes: it can be induced by infection, inflammation and iron loading and suppressed by iron deficiency, hypoxia, and expanded erythropoiesis [50]. We hypothesise this observation is because the typical diet of this cohort of children is low in iron which would impact on iron loading thereby having an impact on hepcidin response.

Within the African American population, rs1800629 in TNF was significantly associated with lower plasma ferritin concentrations. This SNP has previously been associated with iron deficiency as part of a haplotype together with other *TNF* SNPs [51], as well as with an increased risk of severe anemia in low-birth-weight children in Kenya [52] and increased risk of severe malarial anemia in Gambian children [53]. It has been suggested previously that this haplotype would only influence iron status due to pressure of infectious diseases [51] However, our African American cohort consisted of apparently healthy elderly subjects who are not at risk of developing malaria. Nevertheless, there may still have been undetected low grade inflammation from undiagnosed infections or diseases in this elderly population. Disease induced inflammatory processes may therefore underlie this observation.

A limitation inherent to our study is that age and sex distributions differed radically between some of the cohorts analyzed and therefore simple adjustments may not have eliminated all confounding by these variables. In future studies, including cohorts that are more similar in aspects such as age and sex would be desirable. Additionally, our cohorts were smaller than those used in GWA studies and therefore may have lacked power to confirm previously observed associations. Indeed none of the associations with either Hb or SF as outcome measures were significant after FDR correction for multiple testing. The sample size for detecting associations between disease and SNP markers is affected by prevalence of the condition under study, allele frequency, linkage disequilibrium (LD), inheritance models (e.g., additive, dominant, and multiplicative models), and effect size of the genetic variants (e.g., odds ratio, relative risk, etc.) [54–56]. We conducted a retrospective sample size calculation in which we assumed an additive model. From the calculation, 780 individuals are required to conduct association analyses for a SNP explaining 1% of the variation in the outcome measure, with α = 0.05 and power of 80%. If the genetic variant is one of 20 independent genetic variants tested, adjustment for multiple testing using a Bonferroni correction would require a significance level of 0.05/20 or 0.0025 as the threshold. To detect this same association at this adjusted significance level, with 80% power would require a large increase in sample size.

We observed large differences in MAF of investigated SNPs between our study cohorts and those of the Europeans included in the 1000 Genomes project. This may be due to selective pressure caused by environmental conditions thereby altering the frequency of genetic variants between different populations [57]. Theoretically, these differences in MAF between populations could result in dissimilarities in the prevalence of a disease outcome, for example iron deficiency, and in the case of strong gene-disease associations call for population specific strategies to address this.

All of our cohorts were not specifically designed to study the genetics of iron metabolism and therefore not all of the factors taken into account in sample size calculation were considered. However our study does offer an initial indication of what genetic loci would be important for haematological traits in African ancestry cohorts. Another important consideration not taken into account in our analyses is the linkage disequilibrium structure in African ancestry populations, which is different from that of Europeans resulting from the greater variation within the genome of African ancestry populations [58]. It is thus possible that actual disease markers tagged by the investigated SNPs in European populations are not in LD in our African cohorts, and could explain why only a few significant associations were observed. A recent genome wide admixture and association study indeed discovered novel loci to be associated with iron status in an African-American population [59].

In conclusion, we have attempted to replicate reported associations between genetic loci and iron status indicators from European ancestry populations in various African ancestry populations with limited success. This may partly be due to the greater genetic variation within the African ancestry populations [60] which limits the transferability of findings from European ancestry populations. Additionally, populations vary by ethnicity in terms of allele frequencies and biological adaptations due to various environmental factors. For these reasons, population specific genomic studies are warranted. GWA studies in African ancestry populations can potentially reveal novel genetic variants that are specific to these populations. In addition, functional studies are required to further explore the mechanisms involved in the regulation of iron metabolism.

## Supporting Information

S1 Table Associations of single nucleotide polymorphism with iron status parameters. SNP = Single nucleotide polymorphism; Ke = Kenyan cohort; Tz = Tanzanian cohort; SA = South African cohort; AA = African American cohort; P(Het) = P value of heterogeneity testing; Met- = meta analyses of beta and SE values from African cohorts; Met_All = meta analyses of beta and SE values from all cohorts (Afrian+African American); Direction = direction of association based on beta value Hb = Haemoglobin, Values in bold are significant. Results are presented as mean (SE) change per copy of the minor allele from regression analysis using additive genetic model and with adjustment for age and sex for associations involving Hb; and for age, sex and CRP concentrations for associations involving ferritin. ^†^ Values not adjusted for gender as all participants were women. Values in bold are significant before FDR correction for multiple testing. Explained variance ranged from < 1% to 3%, with the largest explained variety being 3% for rs10421768 in the Kenyan population.
(DOC)
