# Supplementary material for: Associations between Common Variants in Iron-Related Genes with Haematological Traits in Populations of African Ancestry
Source: PLoS One. 2016 Jun 22;11(6):e0157996. doi: 10.1371/journal.pone.0157996 (PMC4917107; doi:10.1371/journal.pone.0157996)
Supplement: S1 Table — SNP = Single nucleotide polymorphism; Ke = Kenyan cohort; Tz = Tanzanian cohort; SA = South African cohort; AA = African American cohort; P(Het) = P value of heterogeneity testing; Met- = meta analyses of beta and SE values from African cohorts; Met_All = meta analyses of beta and SE values from all cohorts (Afrian+African American); Direction = direction of association based on beta value Hb = Haemoglobin, Values in bold are significant. Results are presented as mean (SE) change per copy of the minor allele from regression analysis using additive genetic model and with adjustment for age and sex for associations involving Hb; and for age, sex and CRP concentrations for associations involving ferritin. † Values not adjusted for gender as all participants were women. Values in bold are significant before FDR correction for multiple testing. Explained variance ranged from < 1% to 3%, with the largest explained variety being 3% for rs10421768 in the Kenyan population. (DOC) [file pone.0157996.s001.doc]

**Supporting information**

S1 Table: Associations of single nucleotide polymorphism with iron status parameters

|  |  |  |  | **Ke** |  | **Tz** |  | **SA**^†^ |  | **Met_A** |  |  | **AA** |  | **Met_All** |  |  |
| --- | --- | --- | --- | --- | --- | --- | --- | --- | --- | --- | --- | --- | --- | --- | --- | --- | --- |
| **Trait** | **Gene & SNP** | **Chr** | **Minor Allele** | **β (se)** | **P** | **β (se)** | **P** | **β (se)** | **P** | **β (se)** | **P** | **P (Het)** | **β (se)** | **P** | **β (se)** | **P** | **P (Het)** |
| Hb g/dL | ***OPRD1*** |  |  |  |  |  |  |  |  |  |  |  |  |  |  |  |  |
|  | rs482692 | 1 | A | -0.20(0.20) | 0.33 | -2.56(1.33) | 0.06 | 0.09(0.12) | 0.45 | -0.06(0.16) | 0.73 | 0.07 | -0.56(0.28) | 0.05 | -0.22(0.20) | 0.27 | 0.03 |
|  | ***SRPRB*** |  |  |  |  |  |  |  |  |  |  |  |  |  |  |  |  |
|  | rs1830084 | 3 | T | -0.14(0.14) | 0.32 | 0.16(1.04) | 0.88 | -0.18(0.14) | 0.20 | -0.16(0.10) | 0.11 | 0.93 | 0.11(0.16) | 0.47 | -0.08(0.08) | 0.32 | 0.54 |
|  | ***TF*** |  |  |  |  |  |  |  |  |  |  |  |  |  |  |  |  |
|  | rs1358024 | 3 | A | 0.19(0.27) | 0.49 | 3.52(4.68) | 0.45 | 0.87(0.54) | 0.11 | 0.39(0.31) | 0.21 | 0.42 | -0.21(0.36) | 0.57 | 0.17(0.20) | 0.41 | 0.35 |
|  | rs1525892 | 3 | A | 0.12(0.11) | 0.29 | 0.11(0.81) | 0.90 | 0.04(0.12) | 0.74 | 0.08(0.08) | 0.30 | 0.89 | 0.12(0.14) | 0.37 | 0.09(0.07) | 0.18 | 0.96 |
|  | rs1799852 | 3 | A | -0.32(0.18) | 0.07 | 1.64(1.62) | 0.31 | 0.41(0.28) | 0.14 | 0.09(0.36) | 0.81 | 0.05 | 0.05(0.25) | 0.84 | 0.03(0.22) | 0.87 | 0.10 |
|  | rs1867504 | 3 | A | -0.02(0.12) | 0.90 | -0.12(0.82) | 0.88 | -0.06(0.12) | 0.59 | -0.04(0.08) | 0.63 | 0.97 | 0.04(0.14) | 0.75 | -0.02(0.07) | 0.79 | 0.96 |
|  | rs3811647 | 3 | A | 0.08(0.13) | 0.53 | 0.53(0.88) | 0.55 | 0.02(0.11) | 0.83 | 0.05(0.08) | 0.55 | 0.81 | 0.10(0.15) | 0.51 | 0.06(0.07) | 0.40 | 0.92 |
|  | rs3811658 | 3 | T | 0.01(0.13) | 0.95 | 1.57(0.99) | 0.11 | 0.10(0.04) | 0.49 | **0.09(0.04)** | **0.01** | 0.26 | 0.08(0.16) | 0.61 | **0.09(0.04)** | **0.01** | **0.45** |
|  | rs4525863 | 3 | A | 0.17(0.13) | 0.17 | -0.68(0.80) | 0.39 | -0.24(0.14) | 0.09 | -0.07(0.19) | 0.73 | 0.07 | -0.20(0.13) | 0.14 | -0.10(0.13) | 0.43 | 0.09 |
|  | rs7638018 | 3 | G | 0.06(0.12) | 0.63 | 0.34(0.94) | 0.72 | 0.07(0.14) | 0.62 | 0.07(0.09) | 0.46 | 0.96 | 0.18(0.16) | 0.26 | 0.09(0.08) | 0.23 | 0.93 |
|  | ***TNF*** |  |  |  |  |  |  |  |  |  |  |  |  |  |  |  |  |
|  | rs1799964 | 6 | G | 0.04(0.13) | 0.74 | 0.65(0.89) | 0.47 | -0.16(0.13) | 0.21 | -0.05(0.10) | 0.60 | 0.40 | -0.05(0.17) | 0.75 | -0.05(0.08) | 0.52 | 0.61 |
|  | rs1800629 | 6 | A | -0.03(0.18) | 0.86 | 2.02(1.27) | 0.11 | -0.02(0.14) | 0.87 | -0.01(0.11) | 0.94 | 0.28 | 0.18(0.20) | 0.39 | 0.04(0.10) | 0.71 | 0.35 |
|  | ***TFR2*** |  |  |  |  |  |  |  |  |  |  |  |  |  |  |  |  |
|  | rs7385804 | 7 | C | -0.12(0.13) | 0.36 | 1.16(0.85) | 0.17 | 0.09(0.12) | 0.43 | -0.25(0.70) | 0.72 | 0.27 | 0.15(0.14) | 0.28 | 0.05(0.08) | 0.55 | 0.26 |
|  | ***CUBN*** |  |  |  |  |  |  |  |  |  |  |  |  |  |  |  |  |
|  | rs10904850 | 10 | A | **-0.38(0.16)** | **0.02** | 0.91(1.19) | 0.44 | 0.07(0.11) | 0.56 | -0.11(0.22) | 0.62 | 0.05 | 0.03(0.16) | 0.86 | -0.07(0.14) | 0.63 | 0.10 |
|  | ***HAMP*** |  |  |  |  |  |  |  |  |  |  |  |  |  |  |  |  |
|  | rs10421768 | 19 | A | **0.37(0.19)** | **0.01** | 0.46(0.94) | 0.62 | -0.01(0.15) | 0.93 | 0.19(0.18) | 0.27 | 0.17 | 0.14(0.12) | 0.12 | 0.17(0.10) | 0.09 | 0.30 |
|  | ***KIAA1468*** |  |  |  |  |  |  |  |  |  |  |  |  |  |  |  |  |
|  | rs9948708 | 18 | A | 0.03(0.11) | 0.82 | 0.03(0.73) | 0.97 | 0.14(0.1) | 0.18 | 0.09(0.07) | 0.22 | 0.76 | 0.01(0.12) | 0.91 | 0.07(0.06) | 0.28 | 0.83 |
|  | ***TMPRSS6*** |  |  |  |  |  |  |  |  |  |  |  |  |  |  |  |  |
|  | rs1421312 | 22 | G | -0.11(0.10) | 0.27 | 0.09(0.76) | 0.91 | -0.05(0.11) | 0.64 | -0.08(0.07) | 0.27 | 0.90 | -0.14(0.12) | 0.22 | -0.10(0.06) | 0.12 | 0.94 |
|  | rs228918 | 22 | G | 0.14(0.10) | 0.16 | -0.86(0.74) | 0.25 | 0.08(0.10) | 0.44 | 0.10(0.07) | 0.15 | 0.39 | -0.08(0.12) | 0.49 | 0.05(0.06) | 0.37 | 0.31 |
|  | rs2413450 | 22 | A | -0.23(0.12) | 0.10 | -0.65(1.13) | 0.57 | -0.23(0.18) | 0.20 | **-0.23(0.10)** | **0.02** | 0.93 | -0.09(0.14) | 0.55 | **-0.19(0.08)** | **0.02** | **0.84** |
|  | rs4820268 | 22 | G | **-0.28(0.11)** | **0.01** | -0.26(0.87) | 0.76 | -0.05(0.14) | 0.74 | -0.18(0.11) | 0.10 | 0.43 | -0.09(0.13) | 0.48 | **-0.16(0.07)** | **0.04** | **0.55** |
| **Ferritin** µg/L | ***OPRD1*** |  |  |  |  |  |  |  |  |  |  |  |  |  |  |  |  |
|  | rs482692 | 1 | A | -0.95(1.10) | 0.63 | -0.99(1.10) | 0.94 | 1.08(1.08) | 0.45 | -0.27(0.69) | 0.70 | 0.31 | -0.78(1.20) | 0.16 | -0.37(0.56) | 0.50 | 0.47 |
|  | ***SRPRB*** |  |  |  |  |  |  |  |  |  |  |  |  |  |  |  |  |
|  | rs1830084 | 3 | T | -0.95(1.07) | 0.45 | -0.94(1.09) | 0.47 | -1.00(1.09) | 0.99 | -0.96(0.63) | 0.12 | 1.00 | 1.02(1.12) | 0.79 | -0.49(0.55) | 0.37 | 0.50 |
|  | ***TF*** |  |  |  |  |  |  |  |  |  |  |  |  |  |  |  |  |
|  | rs1358024 | 3 | A | -1.00(1.14) | 0.97 | -0.86(1.50) | 0.72 | -0.92(1.32) | 0.76 | -0.96(0.68) | 0.16 | 1.00 | -0.98(1.29) | 0.94 | -0.95(0.65) | 0.14 | 1.00 |
|  | rs1525892 | 3 | A | 1.01(1.06) | 0.80 | 1.05(1.07) | 0.46 | 1.09(1.08) | 0.26 | 1.05(0.62) | 0.09 | 1.00 | 1.10(1.10) | 0.37 | 1.06(0.54) | 0.05 | 1.00 |
|  | rs1799852 | 3 | A | -0.98(1.09) | 0.80 | 1.20(1.15) | 0.18 | **-0.62(1.20)** | **0.01** | -0.15(0.69) | 0.83 | 0.34 | 1.23(1.17) | 0.21 | 0.18(0.60) | 0.76 | 0.37 |
|  | rs1867504 | 3 | A | 1.04(1.06) | 0.54 | 1.05(1.07) | 0.52 | 1.03(1.08) | 0.69 | 1.04(0.62) | 0.09 | 1.00 | 1.05(1.10) | 0.62 | 1.04(0.54) | 0.05 | 1.00 |
|  | rs3811647 | 3 | A | 1.03(1.07) | 0.66 | 1.02(1.07) | 0.85 | 1.10(1.07) | 0.19 | 1.05(0.62) | 0.09 | 1.00 | 1.07(1.10) | 0.45 | 1.05(0.54) | 0.05 | 0.97 |
|  | rs3811658 | 3 | T | 1.05(1.07) | 0.48 | 1.05(1.10) | 0.63 | -1.02(1.09) | 0.82 | 0.36(0.69) | 0.60 | 0.30 | -0.98(1.12) | 0.78 | 0.04(0.59) | 0.95 | 0.32 |
|  | rs4525863 | 3 | A | 1.05(1.06) | 0.18 | 1.08(1.07) | 0.26 | -0.98(1.09) | 0.83 | 0.40(0.67) | 0.55 | 0.30 | -0.98(1.10) | 0.78 | 0.07(0.59) | 0.91 | 0.31 |
|  | rs7638018 | 3 | G | 1.00(1.06) | 0.97 | -0.96(1.10) | 0.62 | 1.00(1.09) | 0.99 | 0.37(0.64) | 0.57 | 0.34 | 1.02(1.12) | 0.76 | 0.52(0.55) | 0.34 | 0.49 |
|  | ***TNF*** |  |  |  |  |  |  |  |  |  |  |  |  |  |  |  |  |
|  | rs1799964 | 6 | G | -0.92(1.06) | 0.17 | -0.99(1.08) | 0.92 | -0.88(1.08) | 0.12 | -0.93(0.62) | 0.13 | 1.00 | 1.05(1.12) | 0.62 | -0.47(0.54) | 0.39 | 0.49 |
|  | rs1800629 | 6 | A | 1.02(1.10) | 0.85 | 1.05(1.12) | 0.62 | 1.03(1.09) | 0.74 | 1.03(0.64) | 0.10 | 1.00 | **-0.76(1.15)** | **0.03** | 0.61(0.56) | 0.27 | 0.60 |
|  | ***TFR2*** |  |  |  |  |  |  |  |  |  |  |  |  |  |  |  |  |
|  | rs7385804 | 7 | C | **0.86(1.07)** | **0.02** | 0.95(1.08) | 0.48 | 1.15(1.07) | 0.06 | 0.99(0.62) | 0.11 | 0.98 | 1.07(1.10) | 0.52 | 1.01(0.54) | 0.06 | 1.00 |
|  | ***CUBN*** |  |  |  |  |  |  |  |  |  |  |  |  |  |  |  |  |
|  | rs10904850 | 10 | A | -0.92(1.09) | 0.31 | 1.02(1.12) | 0.81 | 1.04(1.08) | 0.61 | 0.37(0.66) | 0.57 | 0.34 | 1.07(1.12) | 0.47 | 0.53(0.55) | 0.33 | 0.48 |
|  | ***HAMP*** |  |  |  |  |  |  |  |  |  |  |  |  |  |  |  |  |
|  | rs10421768 | 19 | A | 1.02(1.07) | 0.73 | 1.02(1.10) | 0.72 | 1.06(1.10) | 0.53 | 1.03(0.63) | 0.10 | 1.00 | 1.00(1.12) | 0.99 | 1.02(0.55) | 0.06 | 1.00 |
|  | ***KIAA1468*** |  |  |  |  |  |  |  |  |  |  |  |  |  |  |  |  |
|  | rs9948708 | 18 | A | -0.98(1.05) | 0.73 | 1.02(1.07) | 0.82 | 1.03(1.07) | 0.71 | 0.34(0.68) | 0.62 | 0.30 | -0.91(1.07) | 0.24 | 0.03(0.57) | 0.96 | 0.33 |
|  | ***TMPRSS6*** |  |  |  |  |  |  |  |  |  |  |  |  |  |  |  |  |
|  | rs1421312 | 22 | G | -0.94(1.02) | 0.22 | 1.02(1.07) | 0.68 | 1.01(1.07) | 0.90 | 0.33(0.67) | 0.62 | 0.90 | -0.89(1.07) | 0.15 | 0.03(0.56) | 0.96 | 0.34 |
|  | rs228918 | 22 | G | -0.99(1.04) | 0.90 | 1.05(1.07) | 0.55 | -1.02(1.07) | 0.75 | -0.33(0.68) | 0.63 | 0.32 | 1.10(1.10) | 0.25 | 0.01(0.60) | 0.98 | 0.29 |
|  | rs2413450 | 22 | A | 1.08(1.05) | 0.11 | 1.05(1.10) | 0.73 | 1.02(1.12) | 0.90 | 1.05(0.63) | 0.09 | 1.00 | -0.93(1.10) | 0.49 | 0.56(0.55) | 0.30 | 0.49 |
|  | rs4820268 | 22 | G | -0.99(1.04) | 0.83 | -0.96(1.07) | 0.61 | -0.95(1.09) | 0.60 | -0.97(0.62) | 0.12 | 1.00 | -0.98(1.10) | 0.82 | -0.97(0.54) | 0.07 | 1.00 |
